# “Nerves Need Nourishment”: Advertising Phospho-Energon Pills in Early Twentieth-Century Sweden

**DOI:** 10.1093/jhmas/jrae033

**Published:** 2024-10-01

**Authors:** Lauren Alex O’Hagan, Leif Runefelt

**Affiliations:** Örebro University, Sweden/The Open University, UK; Södertörn University, Sweden

**Keywords:** nervousness, marketing, Phospho-Energon, patent medicine, Sweden, neurasthenia, neurosis, spring lethargy

## Abstract

This paper offers the first case study of Phospho-Energon — an early twentieth-century Swedish patent medicine believed to cure nervousness. Using a large dataset of newspaper advertisements, it explores how the product was presented through scientific and medical language, which drew upon a range of visual and verbal rhetoric to convince consumers of its benefits. It finds that pseudoscientific discourse focusing on self-help was regularly used to sell Phospho-Energon, with consumers warned that their nerves were “not allowed to fail” and required “protection” in order to remain healthy. Furthermore, the “science” supporting this discourse gradually shifted over time as neurosis replaced neurasthenia as a diagnostic category and the concept of spring lethargy became popularised. Overall, this study argues that Phospho-Energon stands as an important example of how partial scientific/medical claims can be used as a rhetorical device to sell products to consumers looking for a quick-fix cure for their perceived mental health conditions.

## Introduction

The late nineteenth century heralded the “Age of Nervousness” in Sweden.^1^ Rapid industrialisation and urbanisation had marked an abrupt shift from the traditional rural way of life, leading to the emergence of a societal discourse on nervousness, anxiety, and fatigue as consequences of a modern urban lifestyle. Such afflictions became grouped under the diagnostic category of “neurasthenia” — a term popularised by American neurologist George Miller Beard and defined as “a disorder of modernity caused by the fast pace of urban life.”^2^ Neurasthenia was believed to affect upper- and middle-class men in particular, being a “prestige disease” caused by busy working lives and active minds.^3^ This condition was quickly capitalised upon by entrepreneurs and marketers who saw opportunities to quell people’s anxieties with new products that claimed to build up their resilience of nerves and protect them from illness.^4^ All manner of potions, pills, and tonics emerged onto the market, each with bold assertions about improving one’s health.

One such product was Phospho-Energon — a pill containing organic phosphorus extracted from calves’ brains — which was launched in Sweden in 1912. Marketed as a “nerve-strengthening, energy-building, strength-giving” wonder cure, Phospho-Energon quickly grew in popularity, with one million pills being produced on a monthly basis by 1925.^5^ For almost four decades, the pills were heavily advertised across the Swedish press, constantly shifting and adapting their messages to fit with evolving understandings of anxiety and how the body was affected, before disappearing in 1953 as Pharmacia — the company behind Phospho-Energon — focused their attention on new products made of dextran instead.

This paper offers the first case study of Phospho-Energon, exploring how this patent medicine was presented through scientific and medical language in advertisements, which drew upon a range of visual and verbal rhetoric to convince consumers of its benefits. In the paper, Phospho-Energon is regarded as a patent medicine because it was not classified as a pharmaceutical when it was launched, yet still claimed to have curative or alleviating effects on nerve disorders. Although the production, sale, and advertising of pharmaceuticals were regulated by Swedish law, health-related products that did not contain listed pharmaceuticals were both free to be sold and marketed and thus were regarded as patent medicines. The advertisements come from *Svenska Dagbladet* — one of Sweden’s most popular newspapers — and were published between 1914 and 1950. They are analysed using (multimodal) critical discourse analysis — a method to uncover how language and other semiotic choices are used to create and convey meaning in texts.^6^ The study finds that pseudoscientific discourse focusing on self-help was regularly used to sell Phospho-Energon, with consumers warned that their nerves were “not allowed to fail” and required “protection” and “nourishment” in order to remain healthy. Furthermore, the “science” supporting this discourse gradually shifted over time as neurosis replaced neurasthenia as a diagnostic category and the language of nerves changed to one of wires and electrical impulse malfunctions rather than based on tubes and hydraulic theory.^7^ The study also identifies “springtime lethargy” as a constant focus throughout Phospho-Energon’s thirty-six years on the market — the season framed as one of despair and exhaustion rather than hope and optimism. Overall, this study argues that Phospho-Energon stands as an important example of how partial scientific/medical claims have been used historically as a rhetorical device to sell products to consumers looking for a quick-fix cure for their perceived mental health conditions. Its findings contribute new knowledge on the historical treatment of mental illness and the relationship between food and wellbeing.

While there have been a wide range of studies carried out on patent medicines generally and on neurasthenia specifically,^8^ less attention has been paid to the sub-category of nerves and how this was exploited commercially. Nerve foods and tonics are briefly mentioned in works by Ben Shephard on soldiers and psychiatrists in twentieth-century Britain, John Crellin and Dennis B. Worthen on the social history of medicines, Denise Maines on almanacs advertising Dr. Chase’s patent medicines, and Jill Kirby on a cultural history of stress in twentieth-century Britain.^9^ However, all of these studies concern the UK or US, thereby offering an Anglocentric view of the phenomenon that overlooks the widespread market for nerve products in other countries, such as Sweden. While some work on nerve tonics has been carried out in a Swedish context by Karin Johannisson, her work has predominantly concerned types of historical diagnoses and their gender-based divide.^10^ Equally, Petteri Pietikainen touches on nerve tonics in his study on the language of nerves in Sweden from 1880 to 1950, but the book’s primary focus is on medicine and science during this period rather than advertising.^11^ Our own previous research has explored the development of nerve foods in Great Britain throughout the late nineteenth and early twentieth centuries,^12^ as well as references to curing neurasthenia in historical Swedish food advertisements.^13^ The current paper builds upon our earlier work by offering a comprehensive exploration of nerve products in the context of Sweden, focusing on a forgotten brand that was once extremely popular. In doing so, it sheds important light on early twentieth-century attitudes to mental health and wellbeing and how these could be turned into lucrative opportunities by a canny growing patent medicine industry.

## Medicalised Modernity: Diagnosing Nervousness in Turn-of-the-Century Sweden

While nervousness has always existed, it was first understood as a clinical condition in the nineteenth century. Nervousness thus became a key component of various new diagnostic categories, including neurasthenia.^14^ Neurasthenia was a type of “heroic” disorder that affected the intellectual classes (particularly men) whose health was strained by the culture of speed in the rapidly industrialising and urbanising Swedish society.^15^ In 1899, a report written by physician Karl Petrén was summarised in the journal *Hälsovännen* (Friend of Health), a popular biweekly magazine on medicine and healthcare. It showed that overexertion was the most common reason for neurasthenia, and that twice as many men as women suffered from the condition. Petrén used *nervositet* (“nervousness”) *nervtrötthet* (“nerve fatigue”) and *nervförslappning* (“nerve slackness”) as synonyms for neurasthenia.^16^ This imprecise terminology, evident also in other articles by physicians at this time, points to what international research has shown: that neurasthenia was a “shifting malady” or a “transient mental illness,” a catch-all term that enabled physicians to give classificatory unity to an existing confusion of nervous conditions.^17^ Thus, through its elastic categorisation of a wide range of symptoms, neurasthenia in Sweden, according to Petteri Pietikainen, occupied a “grey zone or middle ground between a state of full health and severe mental illness,” which left it open for both interpretation and manipulation as Sweden entered a “nervous age.”^18^

Neurasthenia as a concept first appeared in Swedish medical journals in the 1880s, when physicians who had visited Salpêtrière Hospital in France wrote reports of patients who they had encountered. The term was consolidated in 1887, following the establishment of the neurological outpatients clinic (*nervklinik*) at Seraphim Hospital in Stockholm and the pioneering clinical neurological studies of nervousness by Swedish physician, Frithiof Lennmalm. Clinical diagnoses of nervousness in Sweden at this time were influenced by French and German medicine, which saw mental illness as a type of somatic disorder. Nervousness was, therefore, considered to be caused by damage to the central nervous system, which resulted in fatigue and pain, along with an inability to concentrate, headaches, insomnia, depression, excitability, irritability, introspection, and excessive emotion.^19^

Discourses of nervousness became regularly disseminated through the popular press and self-help books, thereby engraining the concept in the public consciousness. *Hälsovännen* published many articles about nerves, while classic books on nervousness were translated into Swedish for the first time (e.g., *On Health and Sick Nerves* by Richard von Krafft-Ebing, *Our Nervous Century* by Paolo Mantegazza, *Nervousness and Culture* by Willy Hellpach, *A Practical Treatise on Nervous Exhaustion* by George Miller Beard).^20^ One of the first popular books on nerve disorders written in Sweden was the writer and theosophist Lucie Lagerbjelke’s *Något om nervositet och naturkrafter* (1899, *Something on Nervousness and the Powers of Nature*), while the first books on nerves written by physicians would come slightly later, such as Max Oker Blom’s *Nervositet och uppfostran* (1903, *Nervousness and Childraising*), Jakob Billström’s *Traumatiska neuroser* (1910, *Traumatic Neurosis*), Kjell-Otto af Klercker’s *Om nervositet hos barn* (1911, *On Children’s Nervousness*), and Poul Carl Bjerre’s *Studier i själsläkekonst* (1914, *Studies in the Art of Healing the Soul*). Nervousness had thus turned into a “contagious diagnosis,” i.e., a medical diagnosis that becomes relatively popular in a short period of time and accepted and embraced by the general population.^21^ It was now a *folksjukdom* — a people’s illness or national malady. The provincial doctor and writer on popular medicine Hjalmar Selldén wrote in *Hälsovännen* in 1895 that nervous people had started to fill the doctors’ waiting rooms and that there existed an exaggerated conception that “the whole nation is vulnerable to nervousness, which threatens to lead the whole population to perdition.”^22^ According to Selldén, as well as other influential Swedish physicians such as Henrik Berg (the most prominent writer on popular medicine in Sweden in the early twentieth century), this democratisation of nervousness was a sign of the modern times. In his broadly used and several times reprinted *Läkarebok* (*The Physician’s Book*), Berg defined nervousness as neither health nor illness but rather an intermediate or transitional state: an increased mental irritability with frequent changes in emotions and moods.^23^

This democratisation and transitional status offered an ideal market for all manner of advice and cures to be put forward. At one end of the spectrum were options promoted by physicians, such as hypnotism, talking therapy, and hydrotherapy, while at the other were more questionable choices such as purported blood purifiers, electro-therapeutic products, food supplements, and health tonics.^24^ These latter products capitalised upon the ongoing discussion of new diseases and ailments created by modern urban life, trying to convince the Swedish public to put their faith into products that were, at best, placebos and at worst, toxic. Placebo or not, the success and sales of such products suggest repeat purchase from consumers, indicating that they could, in fact, be empowering for some and help them gain control over the management of their health.^25^

The democratisation, or perhaps rather normalisation, of nervousness is also evident in the readers’ questions in *Hälsovännen*, answered anonymously by physicians, in which nervousness was a recurring topic. In 1895, hypnotism and rest, preferably in the mountains, were recommended for “anxiety and nervousness”; in 1900, the magazine partly agreed with a reader who asked whether cold showers could be an effective remedy for nervousness. No responding doctor recommended patent medicines as a cure. On the contrary, one reply commented on an unspecified advertisement mentioned by a questioner that “the advertisement about a ‘perfect cure for nervousness’ is obviously intended for extremely ignorant and stupid people.”^26^ From the turn of the century, nervousness was also a recurrent theme in the broader press, for instance in the leading weekly for women, *Svensk Damtidning*.^27^ Pietikäinen suggests that the widespread use of terms such as neurasthenia also arose out of the stigma of mental illness that existed at this time.^28^ It was preferable to be “nervous” than “mentally insane” as this condition could be treated outside of an asylum. Writing in *Förhandlingar* in 1907, the psychiatrist Bror Gadelius stated that, with such terms, relatives could fool themselves into thinking that their loved one did not have a mental health disorder, but rather was suffering from a “nerve illness.”^29^

## Peace in a Package: The Advertising of Nerves in Early Twentieth-Century Sweden

The fact that nervousness had an ambivalent position between illness and health opened up opportunities for advertising various products. The market for patent medicines had emerged in Sweden in the late nineteenth century, as in many other countries.^30^ Since the mid-nineteenth century, the production, sale, and advertising of pharmaceuticals were regulated in Sweden as a privilege for Swedish pharmacists. However, products that were not classified as pharmaceuticals were completely free to be produced, sold, and advertised. It was thus important for companies to avoid having their products classified as pharmaceuticals, while still being allegedly health-promoting and nutritional. It was perfectly legal to advertise such goods, even if they were proven to be ineffective; only those proven to be directly harmful could be banned.^31^ To avoid any critique of the claims put forward in their advertisements, manufacturers described their tonics as “chemical technical” rather than “medical.”^32^ The advertising market was organised in a cartel in the 1910s to have a self-cleaning function, but it did not eliminate advertisements for patent medicines. It was not until 1960 that the medical market was regulated in a way that combated these and not until 1971 that the advertising market was regulated.^33^

At the beginning of the twentieth century, the market was dominated by Sanatogen, manufactured by the German company Bauer since 1898. Sanatogen was advertised in a wide range of Swedish newspapers from the turn of the century.^34^ At this time, Sweden had a vibrant chemical industry producing all sorts of health and beauty products, many of which were remedies for nervous disorders and other similar ailments. They were marketed with a rhetoric that was both nationalistic and scientific. Products had names that tapped into the language of nerves (e.g., Neurasthenin, Nervosin), referred to a scientifically researched content (e.g., Lecin, Biogen), or alluded to the benefits that their consumption could give (e.g., the successful Samarin, referring to the good Samaritan of the Bible). Several manufacturers argued that their product was both domestically manufactured and better than Sanatogen, implying that the consumer could get better nerves while favouring Swedish industry and the Swedish trade balance: “Neurasthenin is Swedish made and fully replaces Sanatogen according to analysis.”^35^

Taking advantage of the supposed causal link between weak nerves and illness, manufacturers claimed that their products restored firmness to shattered nerves and stimulated the production of nerve force.^36^ Furthermore, they strengthened bodily resistance against disease and provided important nourishment to the body.^37^ In both their selected words and images, advertisements drew heavily on scientific references to imply that perfect health was possible, but only if people made the *right* choices (i.e., bought a nerve tonic).^38^ This approach took advantage of the Swedish health ideology of the time, which saw education as essential for turning people into healthy citizens and foregrounded wellbeing as a national responsibility.^39^

Many of the tonics were simply placebos or had no effects at all; others, however, had addictive or dangerous ingredients, such as arsenic, strychnine, morphine, opium, or camphor.^40^ Phospho Energon — the focus of this paper — falls into the former category, as shall be seen below.

## Pharmacia and Phospho-Energon: A Miracle Medicine?

According to a well-established story, a Belgian engineer named C.M. de Kunwald walked into the pharmacy Elgen in Stockholm in 1911 and offered the pharmacist, Gustaf Felix Grönfeldt, the rights to a new miracle drug that cured nervous disorders and other similar problems. The following year, the product was launched under the name Energon; advertisements were placed in the Stockholm newspaper *Dagens Nyheter* in the autumn of 1912.^41^ Grönfeldt left his position as a pharmacist and started the company A.B. Energon, which almost immediately changed its name to A.B. Pharmacia. In turn, it became one of Sweden’s most successful pharmaceutical and biotechnology companies in the twentieth century. The company also produced other products in the early decades, such as the throat and mouth pastilles Paramint and Sodamint for heartburn, but Energon, later Phospho-Energon, was the big sales success and the source of income that laid the foundation for the company’s entire business.

Phospho-Energon was based on an argument put forward by physicians from the end of the nineteenth century: that nervousness was “nerve fatigue” and “nerve slackness,” i.e., the result of mental overexertion. Depletion was a key word. Thus, in 1916, an article in *Hälsovännen* explained that in modern life, “one rapidly depletes oneself and becomes a nervous, discouraged and powerless individual.”^42^ The argument could easily evolve into one that the nerves needed to be energised: nourished in some way. It was an argument that the medical profession did not normally make; doctors rarely recommended medication for nervousness and certainly not in terms of nourishment. Rest, preferably in a seaside or sanatorium environment, was the recurring path to recovery. A nervous 17-year-old’s question to *Hälsovännen* was answered with a simple “Go to Tyringe,” referring to Tyringe Sanatorium, one of Sweden’s more well-established sanatoriums. However, physicians were not automatically negative about agents such as Phospho-Energon. Another question on the same page about whether Phospho-Energon or Ferrol, an iron-based agent, was better was answered with the saying that you cannot compare apples and oranges — both agents could be used successfully for the right problem, in the first case when “the nervous system is depleted.”^43^

What Pharmacia did with Phospho-Energon, however, was to cultivate the nourishment argument. As will be seen, throughout the period of investigation, from the 1910s to the early 1950s, the idea that the individual needed to nourish their nerves was central to the advertising. The only question was how this could best be done. Should it be done by artificial or natural means? Of course, the latter was the answer. This is where Phospho-Energon had its unique selling point on the Swedish market. The active substance in the product was “natural,” that is, taken from the animal kingdom: calves’ brains.

**Figure 1:**
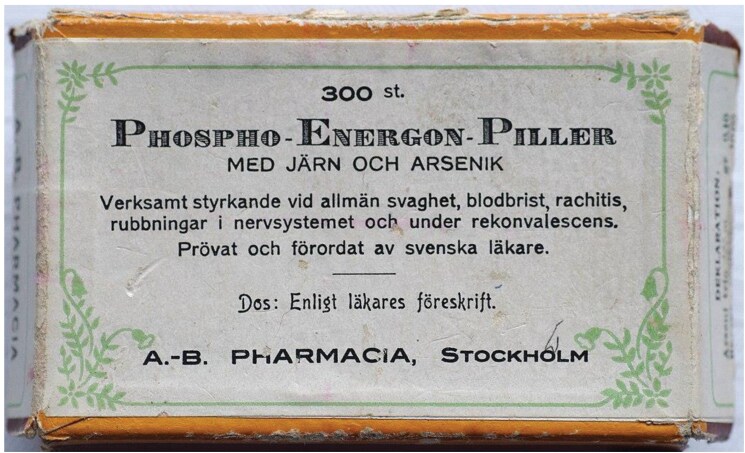
Phospho-Energon CC0 image uploaded to Wikimedia Commons, courtesy of Roland Karlsson.

During the first year, the product was sold as a powder and under the name Energon which referred to energy, i.e., the energy and vigour that the tonic would restore. Other forms of the drug were soon added. In an advertisement in *Dagens Nyheter* from late 1913, Energon maltose, Energon cocoa, Energon chocolate bars and energy pills were all sold, the latter under the name Phospho-Energon.^44^ Over time, the pill, and thus the longer name, came to take over completely (Figure 1). The addition of the prefix Phospho both gave an extra scientific flavour to the product and also anchored it in notions of the benefits of phosphate. There was public opinion in favour of the intake of phosphate as a health benefit. Since the 1890s, other chemical-technical factories had been advertising phosphate powder as necessary for children to avoid rickets, and the press carried articles arguing the benefits of phosphate powder.^45^ The phosphorus supplement may also have been a consequence of Sanatogen’s dominance on the Swedish market. Sanatogen, a milk powder product, was a very different type than Phospho-Energon, but it was enriched with phosphorus, and that supplement was central to Sanatogen’s image as a nerve tonic.^46^

Other already existing tonics on the market such as Lecin were also built around phosphate. In this way, Phospho-Energon was not unique; it was the calves’ brains that made it special and, according to Karin Johannisson, one of the reasons for its success.^47^ Unlike other brands, it was not based on artificially produced phosphate, but rather on organic material. Pharmacia argued that artificial materials were not assimilated by the consumer’s brain substance and thus had little or no effect. The organic material, on the other hand, was readily assimilated, restoring balance to the patient’s brain and nerves.^48^

Johannisson has highlighted Pharmacia’s intensive advertising of Phospho-Energon as a reason for its success, portraying it as deliberately advertising in relation to competitors but also to an emerging criticism of nerve agents in particular and the unregulated market for health products in general in the mid-1910s.^49^ But Phospho-Energon was not heavily promoted in the press between 1914 and 1923. Nonetheless, the tonic seems to have sold well — the company expanded rapidly at this time — and when advertising picked up again in 1923, it had built a brand-new production facility in Liljeholmen, Stockholm. On 27 December 1925, *Svenska Dagbladet* dedicated a one-page article to Pharmacia, offering a behind-the-scenes view into the factory and highlighting Phospho-Energon as a star product. Through images of workers in white lab coats, metallic vats, glass beakers, and machinery, they sought to convey scientific expertise and emphasise Pharmacia’s innovation.^50^ Just over one week later on 8 January 1926, Pharmacia was in the newspaper again, this time in an article promoting a new exhibition of their products — including Phospho-Energon — in *Svenska Dagbladet*’s dispatch office. Phospho-Energon went on to become one of the most commercialised nerve tonics of the interwar period. It was only in 1953 that advertising of the product ceased. By then, Pharmacia had embarked on a new path, launching a range of products based on a different basis than Phospho-Energon, but funded by its revenues.

## “Success Depends on Nerves”: Phospho-Energon as a Cure for Neurasthenia

When nervousness first became highlighted as a serious disease in late nineteenth-century Sweden, women were considered most vulnerable. Heavily embedded within the Swedish ideology of domesticity was the view that women were both mentally and physically fragile, considered to have weaker nervous systems than men, and be particularly vulnerable to nervous disorders. Accordingly, advertisers, the popular press, and medical science all mainly targeted and talked about female nervousness at the turn of the century, although male nervousness was also occasionally highlighted as a problem. This began to change, however, following the new diagnostic category of neurasthenia. Companies such as Pharmacia, who sought to broaden their customer base, saw neurasthenia as the key way to do so. Now, men’s nervousness was just as interesting as women’s.^51^ This was underpinned by physicians, for instance by Karl Petrén, who pointed to men’s overexertion as a prime reason behind neurasthenia and by articles in *Hälsovännen* using a stereotype of the overworked businessman when discussing neurasthenia.^52^

**Figure 2:**
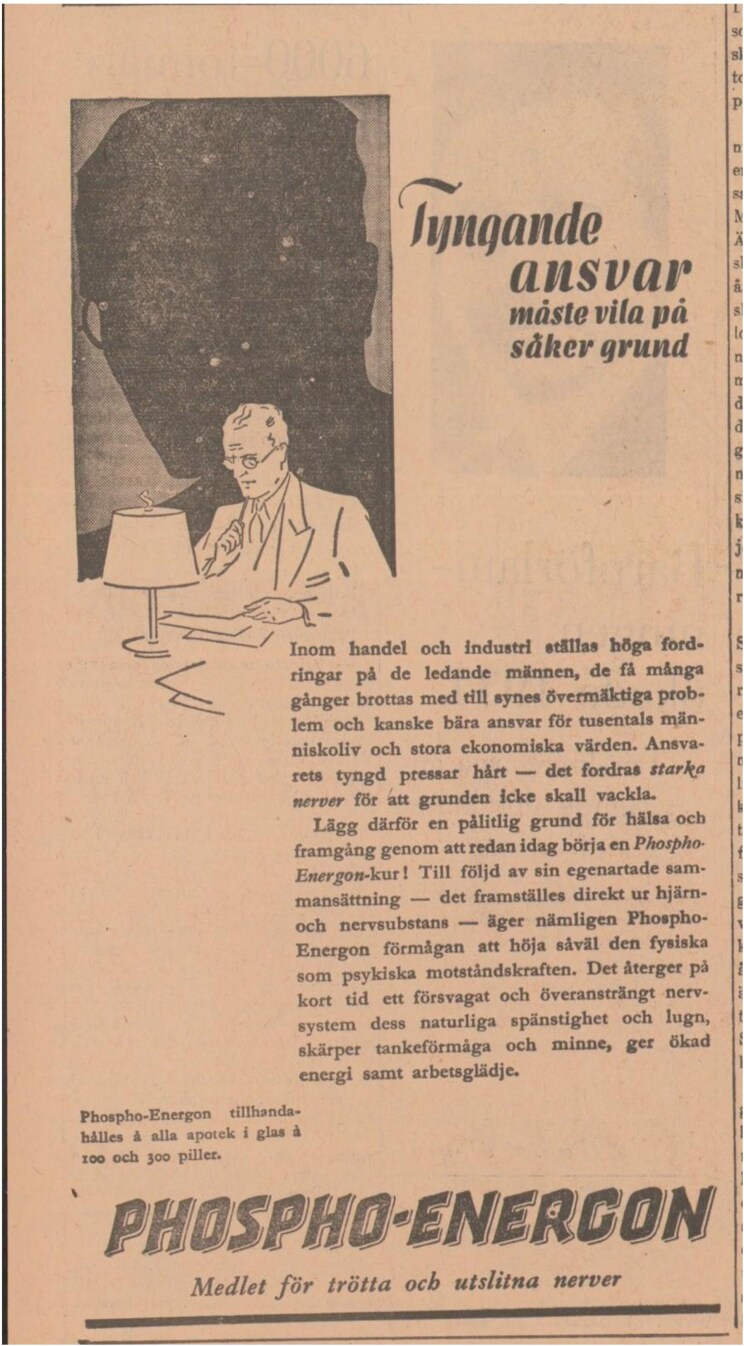
“Onerous Responsibility Must Rest on a Safe Foundation” *Svenska Dagbladet*, 11 February 1935.

Although early Phospho-Energon advertisements do not explicitly state the word “neurasthenia,” it is clear that this loose diagnostic category is at their heart. In line with the belief that this nervous disorder was a prestige disease, intellectual men became the main target of the product. Images show stressed men in their offices shouting into the phone, trying to multitask at their desks, or staying late at night to work through a pile of paperwork (Figure 2). Male university lecturers are also depicted in many advertisements, giving classes to a large group of students or writing on a blackboard. Not only do these jobs imply high status, but it is also connoted through appearance, with most figures being middle-aged, wearing suits, and spectacles — all markers of respect, intellect, and authority.^53^ The symbolic use of black and white is also important here, playing upon notions of good versus evil,^54^ with the contrast serving to depict shadows and a sense of heaviness, gloom, and burden hanging over the person as they attempt to work.^55^

The supporting text also reinforces the idea that neurasthenia only affects male intellectuals, as seen in the following examples, which set up a threatening context:

In trade and industry, high demands are placed on the leading men, the few often struggle with seemingly overwhelming problems and perhaps bear responsibility for thousands of human lives and large financial values. The burden of responsibility presses hard. Strong nerves are required so that the foundation does not falter.^56^

The intellectual worker always notices more strongly than others how the restless pace of modern life, rapidly changing impressions, and idle nervous tension eat away at the nerves and threaten to wear them out prematurely. It becomes harder to keep your thoughts together, to concentrate with full energy on existing tasks, and to give your best.^57^

Words such as “struggle,” “overwhelming,” “burden,” “falter,” “eats away,” and “threatens,” as well as the notion that a mental breakdown could affect “thousands of human lives” and “large financial values,” emphasise the importance of maintaining strong nerves and place responsibility on men to act and protect themselves, i.e., by buying Phospho-Energon.

This sense of pressure was heightened by the use of rhetorical questions that directly addressed men and played to their insecurities: “Have you done too much overtime?” “Rushed by the pace of time?” “Is your ability to work running out?” “Are you holding your nerve?”^58^ Snappy headlines also drew attention to both the dangers of shattered nerves and Phospho-Energon as a helpful remedy: “Priceless for People with Intellectual Jobs,” “Success Depends on Nerves,” and “Onerous Responsibility Must Rest on a Safe Foundation.”^59^ Men were constantly warned that “coming to the realisation late that the nerves have gone wrong is always punishing” and that they must “fight fire with fire” and “equip [themselves] in time to face the pressures before [their] strength runs out.”^60^

**Figure 3:**
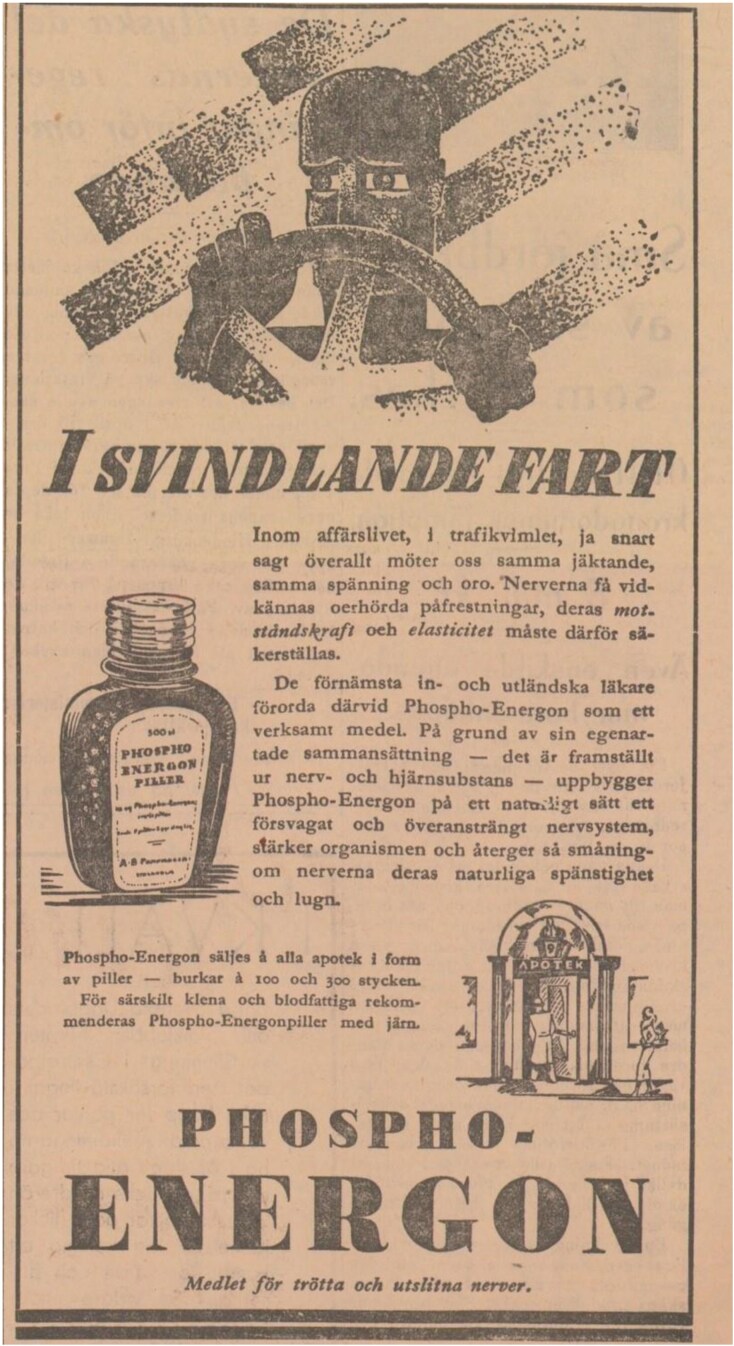
“At Breakneck Speed” *Svenska Dagbladet*, 8 March 1933.

By the early 1930s, advertisements also began to capitalise upon the culture of speed in Sweden with such titles as “At Breakneck Speed,” accompanied by images of stressed men in traffic jams gripping the steering wheels tight (Figure 3).^61^ In 1930s Sweden, there were 187 cars per 1,000 inhabitants, meaning that car ownership was limited to the middle and upper classes.^62^ Thus, such images again served to embed neurasthenia as a male prestige disease. In such advertisements, comparisons were often drawn between the traffic and busy working life: “In business life, in the rush of traffic, pretty much everywhere, we meet the same hustle and bustle, the same tension and anxiety. The nerves are subjected to tremendous stress, their *resilience* and *elasticity* must therefore be ensured.”^63^

Across advertisements, the fast pace of modern life was tapped into through the use of motion lines to underline titles.^64^ Furthermore, a sense of urgency to act was foregrounded by the clever use of font, with words often written in wobbly lettering to accentuate a feeling of nervousness and tension.^65^ Visual cues were also drawn upon to counter these concerns. In some cases, for example, the curlicue of the “P” in Phospho-Energon was depicted as an arrow that pointed directly to an image of the product, thereby creating a directional flow that signalled “causality.”^66^ Additionally, some headings referring to the benefits of the product (e.g. Energy…) had a vertical orientation, the upward motion implying positivity and happiness.^67^

Neurasthenia was seen as a somatic disease that manifested physically; nerves that became “shattered” or “weakened” led to exhaustion, which caused mental breakdown. The prevalent medical orthodoxy of the time — allopathy — furthered the idea that the most effective way to treat neurasthenia was to produce the somatic condition opposite to the pathological state in question.^68^ In other words, by building the body’s strength and resilience, nerves would be safeguarded. In the above examples, Phospho-Energon clearly tapped into this medical discourse. Nerves required constant “nourishment” in order to function properly and that “increased strength and new fresh energy means *health* and *work ability*.”^69^ An often reused headline read, “Even the least effort costs nerve substance,” followed by the claim that the nerves must be fed regularly to work, something which Phospho-Energon did as it was based on natural “brain and nerve substance.”^70^ Such pseudoscientific statements simplified the complex processes at work in the body, somewhat undermining the problems of sufferers and giving them a false sense of hope by claiming that their mental health could be fully restored through Phospho-Energon.

Some advertisements provided more comprehensive explanations of what nerves were and how they worked, explaining the argument about nourishing the nerves:

Every thought, every impulse, every sensation of what is happening and going on around us is mediated by an infinite number of nerve cells, which together form our clever nervous system. But these cells can only fulfil their important function if the substance present in them is sufficient and of such a nature that it gives the nervous system the ability to withstand stresses: if the “nerve substance is depleted,” the most serious diseases can be the result.^71^

The triadic repetition of “every,” coupled with the repeated references to the present (“happening”) and past (“happened”), emphasised the importance of nerve cells to the human body, while the claim that “serious diseases” are the “result” of damage to them built a sense of anxiety and urged readers to act (i.e., by buying Phospho-Energon). The fact that “nerve substance is depleted” carried quotation marks was also significant as it suggested a direct quotation, yet breaking convention, no reference was provided. This left readers to fill in the gaps and assume that this was a quote from a reputable physician or scientist, given the heavy scientific discourse surrounding it. The authority of this particular advertisement was furthered by the fact that it was one long text block with a bold heading, heavily compressed font, and no images, thus mimicking the format of an informative article. This advertisement featured alongside other news articles rather than on a dedicated advertisement page, thereby fusing textual and paratextual information into one and making it easily mistaken for factual content.^72^

Referencing the lasting effects of Phospho-Energon, advertisements frequently called upon the domain of medicine, advising readers to take a “Phospho-Energon cure” or undergo “a course of Phospho-Energon every year.”^73^ Such references implied that the product had been medically approved, while the terms “cure” and “course” were reminiscent of treatment procedures.^74^ In doing so, they built authority and legitimation, positioning the brand as knowledgeable and a worthy equivalent to visiting an actual physician. Potential consumers were also to be reassured by the claim that the product was “clinically tested” by “Inspector Thor Ekecrantz.”^75^ Ekecrantz was indeed a well-known pharmacist and former professor of chemistry and chemical pharmacy at the Pharmaceutical Institute in Stockholm, as well as the founder of the *Swedish Pharmaceutical Journal* in 1897. However, when reaching the age of retirement (he was born in 1856), he left academia for the private sector and was working for Pharmacia as an inspector between 1921 and 1930, and as head of its experimental laboratory from 1923 to 1930.^76^ Having his name connected with Phospho-Energon imbued the product with expert authority, leaving little room for readers to question its efficacy.^77^ Similarly, some advertisements noted that Phospho-Energon was praised by “the most renowned Swedish and foreign doctors.”^78^ Although this statement was extremely vague and potentially fictitious, Phospho-Energon was able to provide a general sense of medical integrity. As Claire Louise Jones notes, such subtleties were often missed by the average reader who saw such information as openness of exchange between brand and consumer that portrayed a unified image of progression and science.^79^ The claim that “thousands of people” have recovered their physical and mental strength with Phospho-Energon worked in a similar way.^80^ The sheer quantity sounded impressive, while also constructing a form of role model authority as the “thousands of people” stood in for the typical everyday contacts who would have provided word-of-mouth testimonials when towns were smaller and populations were less diverse.^81^

## “Stimulate the Nerves and Restore a Sunshine Mood”: Phospho-Energon as the Modern Everyman’s Cure for Weakness

During the 1930s, Phospho-Energon continued to target white-collar men, but at the same time expanded the target audience for its advertisements to include both workers and women. While advertisements aimed at educated men emphasised their great responsibilities and how this strained their nerves, advertisements aimed at working-class men argued that good nerves were essential to maintaining one’s ability to work. This is apparent in a 1933 advertisement which showed a worker with a factory and some large cogwheels in the background. The turning cogwheels symbolised the worker’s importance as a cog in the modern system,^82^ but they also alluded to the notion of the body as a machine in need of being carefully “oiled” to function properly.^83^

**Figure 4:**
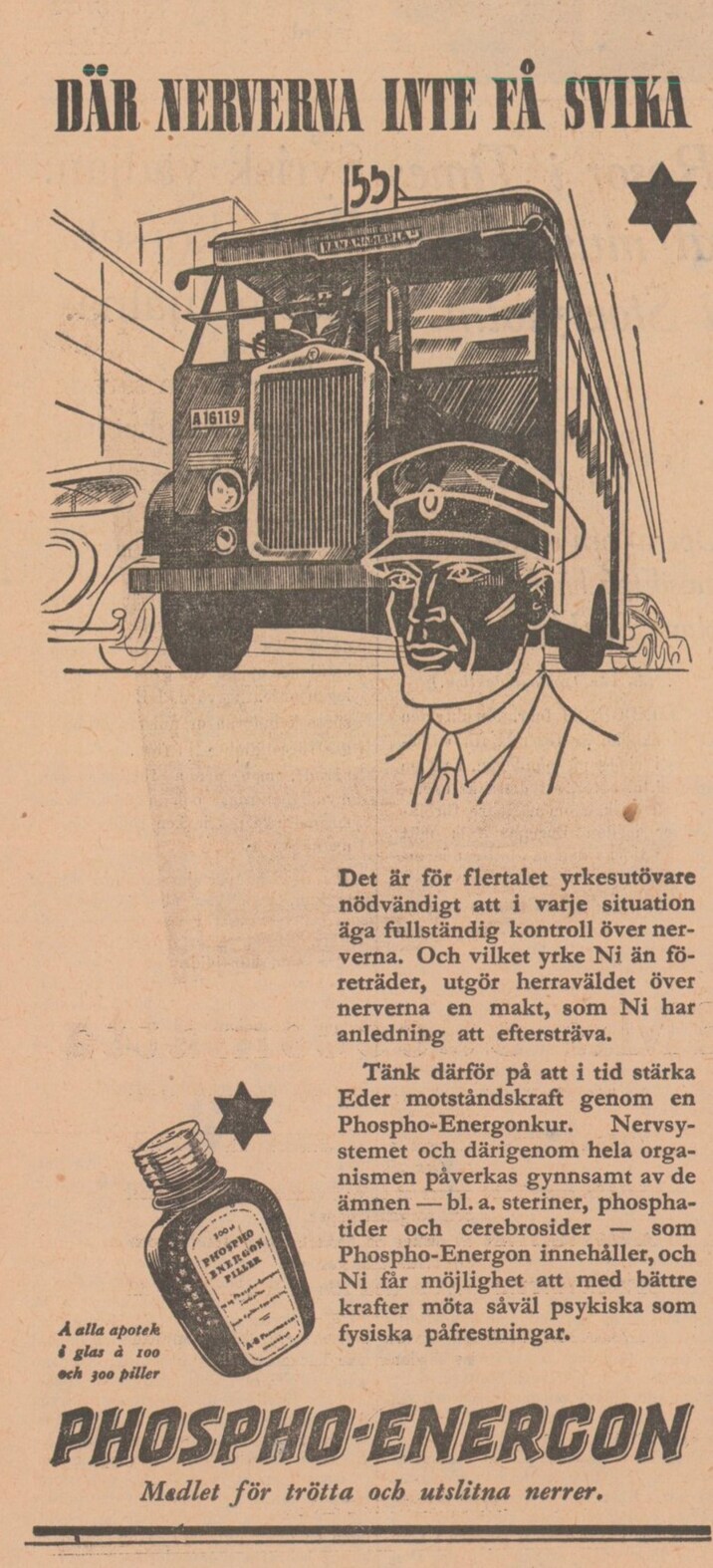
“Where Nerves Are Not Allowed to Fail” *Svenska Dagbladet*, 24 February 1936.

In a similar vein, a 1936 campaign depicted traffic policemen and bus drivers — workers with stressful, modern jobs associated with the fast pace of modernity.^84^ Using the heading “Where Nerves Are Not Allowed to Fail” (Figure 4), these advertisements made men feel guilty and personally culpable if they did not protect their mental health, thereby implying a certain irresponsibility and selfishness for those who conceded to weakness.^85^ The advertisement in Figure 4 showed a bus driver on the 55 route — a real Stockholm bus route that started at Vanadislunden. This presented a clear location with which many readers might be familiar and, therefore, offered a relatable slice-of-life scene. The accompanying text framed mental health as a battle in which one must become victorious: “It is necessary for the majority of professionals to have complete control over their nerves in every situation. And whatever your profession, mastery over the nerves is a power to which you have reason to aspire.” Here, being in control of one’s mental health and wellbeing was framed as a skill that could supposedly be acquired. Furthermore, to acquire this skill was a duty, and not to do so was to let oneself down. This notion of personal responsibility fit strongly with the Swedish health ideology of the period, whereby being a strong citizen was seen as one’s moral duty and to neglect health through poor (nutritional) choices was deemed selfish and inexcusable.^86^

Likewise, the company now targeted a female audience and depicted women in its advertisements. According to Phospho-Energon, modern life, with its speed and high demands, was also a strain on women’s nerves, which needed constant nourishment. An advertisement from 1934 showed a woman sleeping peacefully, contrasted with a text that focused on the stress of everyday life. This was emphasised by the heading “Five Quarters of an Hour,” which implied the need for extra time in the day to complete all the accumulating tasks.^87^ The accompanying text stated that a daily morning intake of Phospho-Energon could ensure a good night’s sleep after a hard day’s work and make sure that you woke up feeling fresh and ready to go the next morning. While women in Phospho-Energon advertisements were still rare in 1934, they were regularly featured in campaigns from 1937 onwards, just as much as men. This was likely due to the increasing number of working women (representing approximately 27% of the total workforce at this time), supported further by the Social Democratic rise to power in 1936, which promoted a form of “welfare feminism.”^88^ In line with this, photographs showed women in various roles that extended beyond their duties as mothers and housewives, such as typists, maids, and shop assistants.^89^

It seems that the advertisements generally reflected Swedish urban industrialisation, thus underlining the extent to which nerve diseases were the consequence of modern life. Farmers were one group missing from the advertisements; it was industrial workers, housewives and the growing number of professional women in the urban service sector that they depicted. In doing so, they joined an old tradition, as advocated in *Hälsovännen*, that fresh air, manual labour, and working with your hands in the soil were a cure for nervousness — and also a reason why nervous symptoms had been so uncommon in the old peasant society.^90^ The strategy emphasised the need for modern urban women and men to nourish their nerves.

While the widening of the target groups partially stemmed from a desire for a larger customer base, it was also influenced by changes in the general view of nerve disorders, away from neurasthenia towards a more general, vaguely defined nerve weakness. The message was that weak nerves could affect anyone who lived an intense life, without any link to more basic mental disorders or diseases. The advertisements normalised nerve weakness, which took the same form in anyone who became overworked: tiredness, lack of willpower, bad temper. This normalisation of nerve weakness extended more broadly in Swedish society through Swedish Radio broadcasts aimed at enlightening and educating the general public on health matters. Most notably, a lecture series by psychotherapist Iwan Brett that ran throughout the 1930s received widespread attention.^91^ However, this normalisation was not received positively by all. Psychiatrist Victor Wigert complained in a 1932 article for *Social-Medicinsk Tidskrift* about bookshop tables “filled with scientific and popular books on ‘neuroses’” and the concentration of “violent energy on psychopathological phenomena” in both novels and theatre productions.^92^

Phospho-Energon advertisements increasingly emphasised moods and atmospheres, along with the need not only to cope but also to feel joy during the working day. This vaguer view of nerve disorders was in line with the use of the term “neurosis” in the 1930s, but the advertisements avoided that word. Neurosis as a diagnosis was not considered physical because it did not indicate any demonstrable anatomical deterioration of nerve tissues.^93^ This approach was incompatible with the basic idea of Phospho-Energon: that nerves were anatomically weakened if they were not nourished. Thus, Phospho-Energon’s persisting focus on nerve weakening served to play down the stigma of mental illness by equating signs of nervousness more with a physical, rather than a psychological, condition. In doing so, it echoed earlier words by psychiatrist Bror Gadelius around references to nervousness, as opposed to lunacy, being strongly influenced by “prejudices about the aberrant nature of mental illness.”^94^ This idea remained at the heart of the product, and advertisements from 1935 onwards typically included a recurring sentence that broke down the mysterious nature of Phospho-Energon by naming specific components and why they were needed: “a compound of Sterines, Phosphatides and Cerebrosides,” allegedly coming from calves’ brains, that the brain and nerves needed in order not to weaken.^95^

Sterines are cholesterol-like compounds found naturally in many plant-based foods, phosphatides are fatlike, phosphorus-containing substances, and cerebrosides are important components of animal muscle and nerve cell membranes. It is highly unlikely that consumers understood any of this heavy scientific jargon; nonetheless, in outlining the ingredients of Phospho-Energon, the brand connoted transparency and honesty, directly responding to concerns that many commercial nerve remedies were either placebos or toxic in nature.^96^ Consumers were informed that nerves were “*dependent*” on access to these three energy substances, thereby suggesting that not to take Phospho-Energon would result in a depletion of such resources. In some advertisements, these components were even described as “active ingredients,” reflecting an early use of a term seen so frequently in twenty-first-century advertising.^97^ Again, this embedded the product in medical discourse as active ingredients are the drug substance in pharmaceuticals. However, Phospho-Energon provided no details of the excipients (i.e., the pharmaceutically inert materials of the pills); without this information, consumers lacked knowledge of how effectively the active ingredients could actually reach the target site in the body.^98^

As part of the normalisation effort, *nervousness* no longer had to be the core part of the argument, or even an argument at all. In the campaign carried out in 1937 under the heading “Healthier than most!” previously used words such as “nervousness” and “nerve weakness” had disappeared altogether. Instead, Phospho-Energon was presented more as a general pep pill that counteracted fatigue, lack of willpower, and lethargy. The advertisements emphasised the importance of “starting in time,” i.e., taking Phospho-Energon as soon as possible, preferably before one had even become tired and weak.^99^ Indeed, according to one of many advertisements in the autumn of 1937, one should take the pill as “protection against psychological stresses of various kinds,” even when fully healthy, and that “you may often be able to determine in advance when the workload will be particularly heavy,” so a treatment could be started as a precautionary measure.^100^ Phospho-Energon was now just as much *preventive* as *reactive*, thereby building its new image as an everyday product necessary to keep fit and healthy. The pills were also highly convenient, could fit easily in one’s pocket or handbag, and be taken at any time when the need or desire arose, fitting perfectly into one’s busy daily lifestyle.

Pharmacia also began to emphasise modernity in a different way than it had done before with cars and high speed. Now, Phospho-Energon was reputed not just to retain energy for modern working life, but also for something else that characterised modernity and the progress of the emerging welfare state: leisure. Even at leisure, men were considered unsafe and warned not to let down their guard. Advertisements often showed idyllic scenes of walking in the park or skiing, with accompanying text stating how a “day well spent out in nature leaves a feeling of peace and wellbeing.”^101^ This text was then abruptly interrupted with a bombshell: the ability to “make the most of your day” is not “universally given” and, if not careful, “nerve substance [will be] used up faster than it has had time to be newly formed.”^102^ Phospho-Energon was then, of course, presented as a solution to this problem, with its abilities explained simplistically to reader: it helps “overcome weakness,” gives “energy and self-confidence,” and builds “natural resilience.”^103^

The emphasis on the natural was particularly important. At this time in Sweden, the Physical Culture Movement (PCM), which saw healthy bodies as an “obligation of citizenship,” was in full swing.^104^ The PCM called for a return to pre-industrial back-to-basics way of living, and part of this entailed eating well and keeping fit.^105^ Many food manufacturers drew upon nature in their advertisements to try to distance themselves from the complex production processes involved in making their products. Doing so not only kept a mythical aura around the products and their supposed abilities, but also tapped into broader associations of the natural with purity, cleanliness, and unadulteration. Applied to Phospho-Energon, such rhetoric served to frame the product as free from artificial ingredients and working in harmony with the body to ensure good long-term mental health.

Another advertisement in *Svenska Dagbladet* in 1937 showed a portrait of a smiling man, under which it was stated that “Your strength and good mood should not only be sufficient for the working hours — it is also important to have *something left over*, namely for your own part of the day, for your *leisure time*.” Here too, readers were encouraged to start early and preferably “today,” whether or not their strength had begun to fail.^106^ By telling consumers not just about its medical benefits, but also connoting that it was part of a modern lifestyle, Phospho-Energon became incorporated into a daily consumerist lifestyle, growing into a trendy and popular brand consumed by Swedes as part of a ritualised practice.

**Figure 5:**
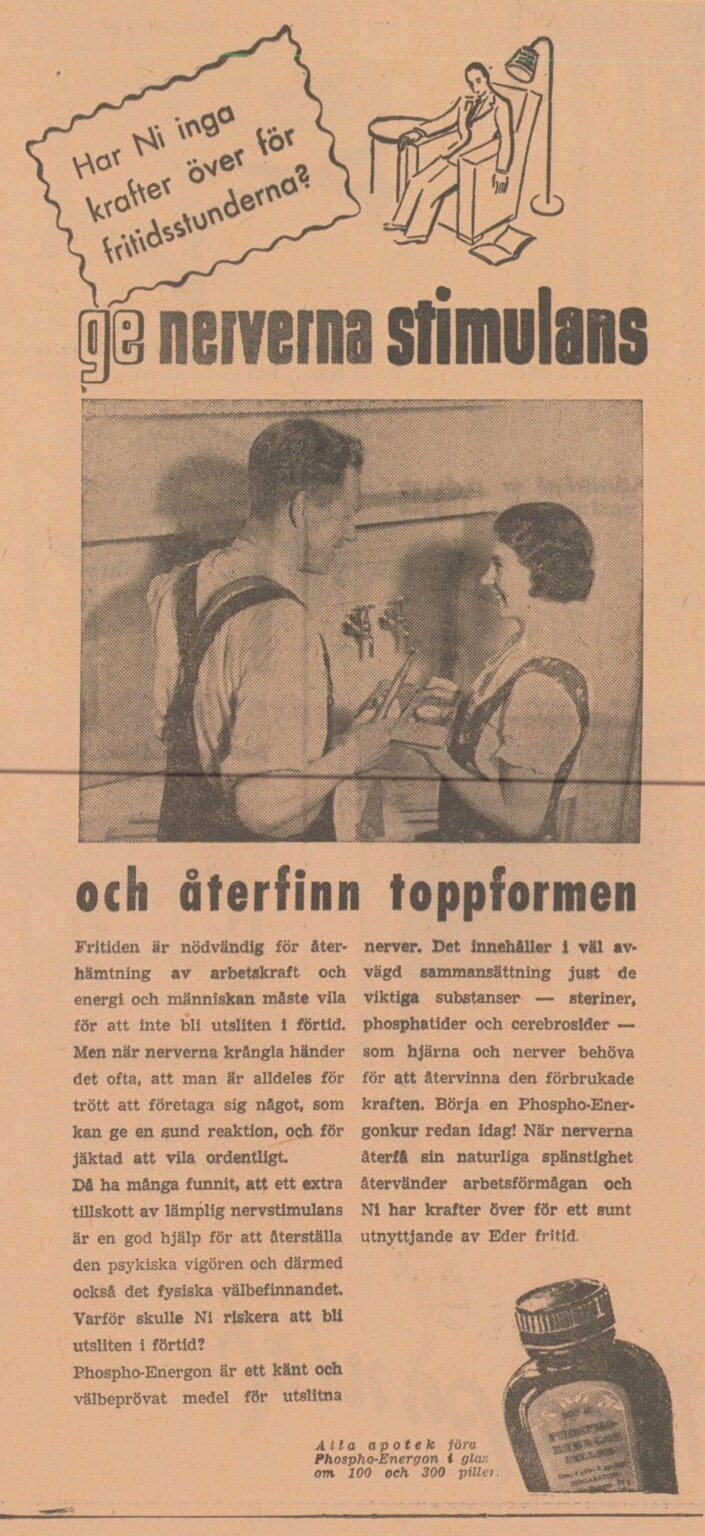
“Give Your Nerves a Boost and Get in Top Shape” *Svenska Dagbladet*, 25 October 1938.

Likewise, an advertisement from the autumn of 1938 showed a married couple working together on a home renovation project (Figure 5). The text explained that leisure time was important for recovery before work, but that it *should* and *could* be more than that; it should also be meaningful. For this, Phospho-Energon was needed.^107^ This message was in response to the recent introduction of Sweden’s first Annual Leave Act, which granted every working Swede the right to two weeks’ paid holiday.^108^ The Social Democrats problematised leisure time, telling the public not to waste their holidays by being idle. These messages swiftly carried over into advertisements.^109^ Likewise, an early advertisement in 1939 read: “Get fit for leisure.” With two weeks of summer vacation ahead, it was wise to start a Phospho-Energon cure in good time — as early as February — so that the two precious weeks would not be lost to fatigue and lack of willpower.^110^

## “Dull in the spring sun?” Phospho-Energon as a Cure for Springtime Lethargy

As seen above, by the late 1930s, nervousness could be irrelevant in Phospho-Energon advertisements, such as in the 1937 campaign. Instead, there was a predominant focus on tiredness and the need to fight weakness through a regular course of Phospho-Energon pills. However, arguments focusing on the need to strengthen the nerves were still valid when the advertisements promoted Phospho-Energon as a remedy for springtime lethargy.^111^

Springtime lethargy (in Swedish, *vårtrötthet* or “spring fatigue”) was an expression that first appeared in Swedish at the turn of the twentieth century.^112^ It can also be found in popular discourse of the time in other Northern European countries, such as the United Kingdom, Germany, and Denmark, suggesting that the condition arose as a consequence of long, dark winters. Today, there is a popular medical discussion about springtime lethargy around the Western world, but medical science takes little interest in it.^113^ While the exact cause of springtime lethargy is unknown, it is typically associated with changes in hormones, blood pressure, and nutrition. Interactions between high levels of melatonin and serotonin stress the body, rising temperatures affect body temperature, while a deficiency in certain vitamins and minerals cause fatigue. This is exacerbated further by daylight saving time, which can disrupt circadian rhythms.^114^

According to an article in *Hälsovännen* in 1899, springtime lethargy was said to be due to anaemia and blood deficiency, but there were several different explanations, none of them satisfactory, as to why these conditions caused renewed and worsened symptoms in the spring.^115^ Early twentieth-century newspaper articles often reported on cases of springtime lethargy and advised readers on what they could do to reduce its impact, yet these discussions frequently lacked any scientific rationale.^116^ In the 1920s, the concept became well established in Sweden, which may have been due to an increase in the range of particular foods and supplements targeting spring fatigue, such as Askamin and Idozan. In 1926, Pharmacia advertised Phospho-Energon as a remedy for springtime lethargy, but otherwise the theme was long downplayed in the company’s advertising.^117^

The first advertisement focused wholly on springtime lethargy appeared on 27 March 1934, setting the scene for what was to come over the next decade. Entitled “An Important Decision for Health and Work Ability” and emphasised by an image of a judge’s gavel slamming down, it directly addressed consumers, beseeching them to recall last spring when “you felt tired and down, your nerves were often weak and every effort took the utmost from your strength.” The assertive language left little room for manoeuvre, acted as “reliable guides to the truth,”^118^ and took as given that all readers suffered from these afflictions last spring. The advertisement continued in the same manner, reminding readers that this was a “costly experience” and asking whether they “dare to risk a repeat this year.” Its heavy value-laden language evoked a sense of fear, directly challenging readers to take better care of themselves, in line with the self-help agenda of the period. Phospho-Energon was then, of course, put forward as a way to protect one’s “physical and mental resilience.” The same advertisement ran for two years with minor variations to its heading. “Spring is coming!” was frequently employed — an innocuous statement that took on sinister undertones in this context.

Other Phospho-Energon advertisements likewise aimed to problematise spring and turn a potentially enjoyable time of year into one of distress and apprehension. This was apparent in an advertisement from 14 April 1934, which showed a silhouette of a naked man with his arms wide open as he fully embraced the rays of the sun. The accompanying headline stated, “You want to enjoy the lovely spring and summer to the fullest…”, which evoked feelings of happiness and freedom. However, expectation was broken with the following incongruent text^119^ that painted a frightening picture of the spring: “How often during those cold, dark winter days have you longed for the spring and the warm, sunny days? But when spring and summer come, you perhaps become deeply disappointed. You are tired and down, that otherwise so lovely and lifegiving sun just irritates you, your sleep is bad, your desire to work and your mood hit rock bottom...” The advertisement went on to emphasise that these were all signs of “shattered nerves” and that something was needed to maintain good health. Framing itself as a compassionate brand, Phospho-Energon recognised that people might not have the time “to take longer leave from work and submit to appropriate medical care.” That was why their product offered “the most rational way” to get better as it could be slotted into everyday life, offering a fuss-free way to “meet spring and summer with new fresh energy.” “Rational” was a buzzword of the era, often carrying reforming and moralistic undertones and used to suggest a respectable social order and culturally harmonious society.^120^ Likewise, the notion of convenience was a popular advertising strategy of the time, used to showcase how a product could enable users to continue their daily activities without interruptions.^121^

**Figure 6:**
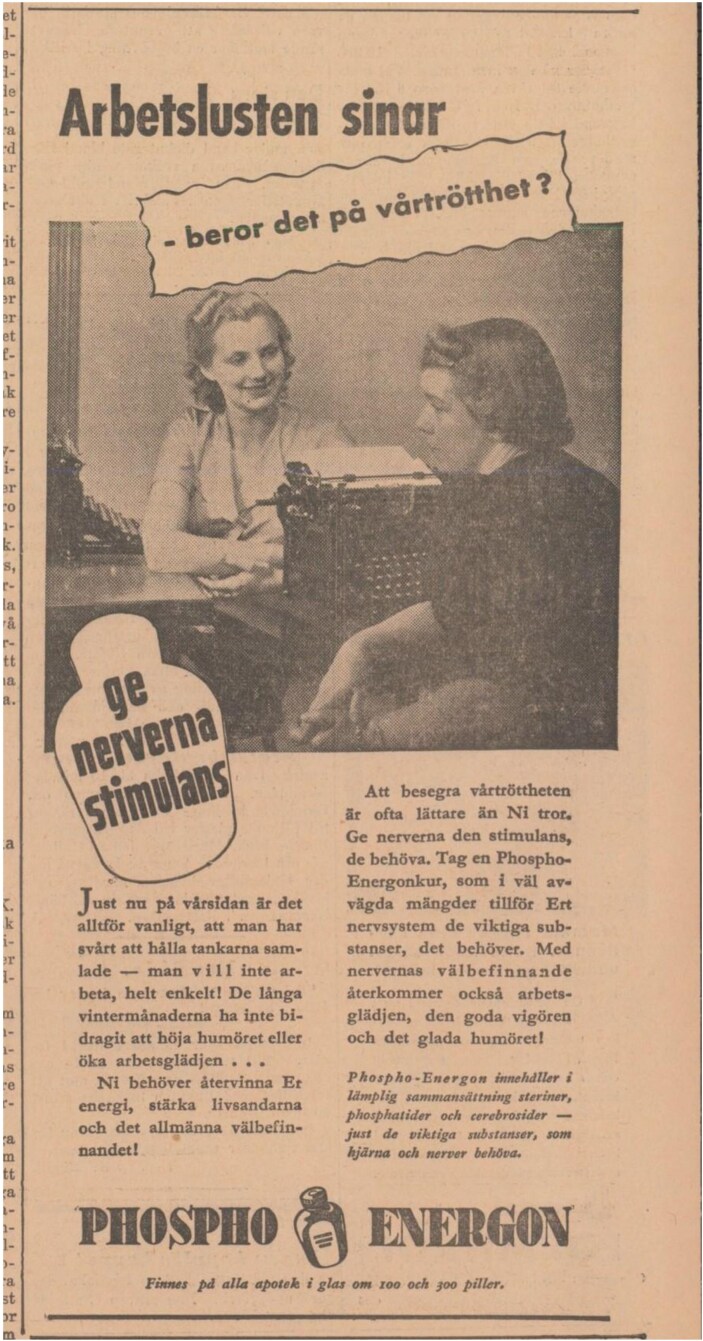
“Does your desire to work depend on spring lethargy?” *Svenska Dagbladet*, 18 April 1939.

Advertisements were also replete with rhetorical questions that spoke directly to consumers, making a link between springtime lethargy and a particular emotion: “springtime lethargy and gloomy?”; “springtime lethargy and pessimistic?”; “springtime lethargy and listlessness?” In later advertisements, these types of statements were accompanied by photographs rather than illustrations. The decision to use photographs created high modality, symbolising reality over fantasy and evoking a more emotional response from consumers than an illustration as they could identify with the figures that they saw.^122^ In these cases, the headlines tended to overlap the photographs, making it clear that they were to be interpreted as connected.^123^ This was the case with an advertisement from 18 April 1939 (Figure 6), which showed two young women at their typewriters alongside the headline, “Does your desire to work depend on springtime lethargy?” The question was written in a box with a wobbly border, chosen deliberately for its metaphorical associations with nervousness.^124^ Its spatial location above one of the women’s heads suggested that it was direct speech from her to her friend. This was furthered by the fact that the friend looked very downcast, her side profile revealing slumped shoulders, interlocked hands, and furrowed brow. Also overlapping the image was the silhouette of a pill bottle with “stimulate your nerves” written inside. Its intrusion onto the photograph established a bleeding of meaning, creating a causal link with the headline and visually implying that Phospho-Energon could solve this medical complaint.^125^ The verbal text reinstated the visual message, outlining that these feelings were “all too common” in spring, but that it was “easier than you think” to overcome springtime lethargy. By creating what Roland Barthes called an anchorage, the viewer engaged with the image first and then used the text to fix a more definite and precise understanding, thereby strengthening the impact of the advertisement’s message.^126^

A similar series of advertisements used both illustrations and photographs to conjure up a “before” and “after” scenario. In all cases, the “before” depicted a negative scene in illustrated form, while the “after” showed a positive outcome in photographic form. The choice to use different types of visuals served to strengthen the reality of taking Phospho-Energon and, therefore, increased the truthfulness and reliability of its claims.^127^ For example, one advertisement showed a businessman at his desk with thoughts swirling around his head contrasted with him speaking happily on the phone; an architect throwing away his set square and paper in frustration contrasted with him smiling as he held up a compass in front of a planning board; and a shopkeeper refusing to help a customer contrasted with him willingly helping her to find a product. In all cases, the question “who doesn’t recognise these typical symptoms in springtime?”^128^ was asked, which served to create a sense of camaraderie and national belonging and set forth combatting springtime lethargy as a collective mission. This fit strongly with the Swedish health ideology of the time, which saw good health as a duty of all citizens to safeguard the country’s future.^129^ These ideas were further accentuated through such statements as “thousands feel tired and down” and encouragement not to “think that it must be like that in spring.”^130^

Militarised language was also heavily employed throughout advertisements, with readers warned to “be on your guard” and get ready to “fight and drive away” illness, “hit hard,” and stop listlessness from “taking over.”^131^ Militarised language and illness have a long history in discourse and have been used in advertising since at least the mid-nineteenth century to promote infection as a predator that would prey upon the hunted (i.e., consumers).^132^ In her study of stress in twentieth-century Britain, Jill Kirby found that such combative language was used to conceptualise nervous disorders as “potentially overwhelming, as something which needed to be ‘outwitted’ or ‘safeguarded’ against and which required vigilance and self-control.”^133^ In a similar vein, the language used by Phospho-Energon presented the medicine as a dutiful protector.^134^ Similarly, springtime lethargy was personified as something “sneaky” that “darkens your whole existence.”^135^ Imbuing this abstract concept with human attributes made it appear scarier.

From 1940, there was a sudden emphasis on iron in Phospho Energon advertisements after the product added the mineral to its pills. The addition of iron to Phospho-Energon tied in with accelerated interest in “nutri-quantification” and the recognition of protective nutrients required for normal body functioning and to prevent nutrient deficiency diseases.^136^ Iron had long been used as an ingredient in patent medicines for anaemia, such as in Vilton’s blood strength preparation, Fercao (cocoa with iron), and the aforementioned Ferral preparation. However, the growing nutritional research on the importance of vitamins for the human body from the 1910s and the knowledge of how to fortify foods with vitamins, vital also in Sweden, led among other things to an official report by the Swedish government in 1937 and to research and actions regarding fortification of foods.^137^ Selected staple foods in Sweden, such as bread, cereal, fat spreads, milk, juice, and salt, became fortified with micronutrients and marketed as staving off single-nutrient deficiency diseases. These new advertising campaigns emphasised the vitamin or mineral content of products and their protective nature, thereby convincing consumers of their necessity. It was an international movement. At the same time as Phospho-Energon received its iron additive, government projects for iron fortification of wheat flour began in both the UK and the US.^138^ However, Phospho-Energon often employed pseudoscience to make its claims. In an advertisement dated 30 March 1942, for example, it warned customers that springtime lethargy was caused by the fact that “a normal supply of iron [is] not found in winter.” Although there is no medical evidence to support this statement, it sounds convincing, particularly when it was backed up by the rationale that the body’s reserves were depleted in winter, meaning that one “cannot truly enjoy the spring.”

**Figure 7:**
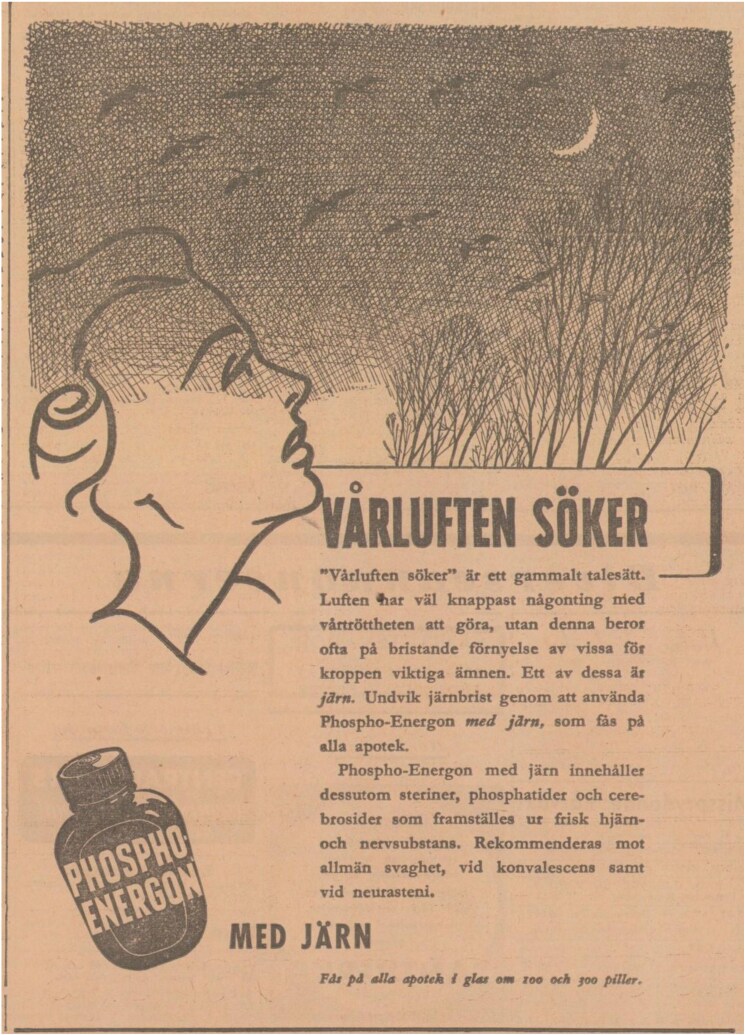
“The spring searches…” *Svenska Dagbladet*, 4 April 1942.

Similar forces were at work in an advertisement entitled “The spring air searches” which, according to the accompanying text, was a traditional Swedish saying.^139^ The phrase can be found in the works of eighteenth-century theologian and scientist Emanuel Swedenborg and the 1871 children’s book *I skogen* [*In the Forest*] by Wendela Hebbe and is used to signify that the air in spring can be tiring.^140^ The advertisement emphasised this point through a side view of a woman with her gaze turned upwards towards the sky (Figure 7). The scene was rather ominous with its crescent moon, dark sky, moving trees, and flock of birds that appeared bat-like. The top of her head was embedded into the black sky, casting her in shadow and implying that a metaphorical shadow hung over her mind.^141^ According to the text, while the traditional saying might not be true, it was true that spring brought “a lack of renewal of certain tissues important to the body, including iron.” Then, it called directly upon consumers to “avoid a lack of iron by using Phospho-Energon with iron.” Another advertisement also made a similar false claim, warning that, “The dark season often uses what the light has brought. When the storage in the body is no longer renewed, it gives a signal. A signal of springtime lethargy, which often means a lack of iron.”^142^ Again, there was no medical evidence to support these statements, but it cleverly tapped into the public’s growing interest in nutrients, framing Phospho-Energon as a reassuring product that could address this new national concern.

## Concluding Discussion

Regardless of the condition that Phospho-Energon could supposedly cure, be it neurasthenia, weakness, or springtime lethargy, all advertisements drew upon similar strategies, using rhetorical questions, militaristic language, personification of illness, triadic structures, buzzwords, pseudoscientific explanations, and specialised medical vocabulary to convince consumers of the product’s effectivity. Advertisements also underscored the importance of keeping healthy, placing responsibility on individuals to engage in self-care regimes for the good of themselves and the nation. Overcoming nervousness, thus, became framed as a national duty, with individuals encouraged to learn the skill of being in control of their mental health. Across advertisements, nature was also emphasised, in accordance with the Physical Culture Movement’s desire to return to a simpler time. In other words, modern life had upset the body’s natural harmony, but Phospho-Energon had the power to restore this balance.

In her study of British patent medicines, Erin Elizabeth Bramwell shows that mothers were not tricked into taking them; rather, they acted deliberately and saw them as one of several ways to do their best for their children.^143^ No similar studies on patent medicines have been conducted in the Swedish context, but it is conceivable that the same arguments could be valid. After all, despite scepticism from doctors and the government about excessive and misleading advertising, there was no substantial criticism either of nutritional and health-promoting products in general, or of Phospho-Energon in particular. Phospho-Energon may have fulfilled the same function as dietary supplements today, products many of us take with some hope but without demanding any real scientific basis for them. Phospho-Energon provided an easily attainable and inexpensive solution for nervous disorders, enabling all classes of society to receive help (or at least believe they were receiving help) for their condition. Coupled with this were concerns about being lazy and not fulfilling one’s moral duty of being fit for the good of the nation. Finally, as the products and its associated advertising remained so loosely regulated, consumers may have assumed that it was a legitimate and safe remedy.

However, starting with a government bill in 1934 on stricter regulation of the pharmaceutical market, gradual measures were taken that made it more difficult to produce and sell patent medicines and nutraceuticals unhindered. In 1941, a special board for the regulation of pharmaceuticals was formed, which had both clearance and policing functions (i.e., to check proposed advertisements and to ensure that published advertisements were not misleading). Perhaps unsurprisingly, from this period onwards, Phospho-Energon advertisements decreased substantially. In the early 1950s, there was a short-lived re-emergence in response to new medical knowledge on the role of choline in the body and its importance for the development of normal brain functioning.^144^ Suddenly, Phospho-Energon advertisements mentioned the presence of choline in the product and its importance for the body, stressing that taking three pills daily “provides the equivalent of one third of choline found in normal food.”^145^ However, after just three years, the product had disappeared entirely from the market. Petteri Pietikainen suggests that the disappearance of nerve products was due in large part to the creation of *Folkhemmet* by the Social Democrats, meaning that the State became more active in efforts to support those with nervous disorders and there was less need to rely on tonics and pills.^146^

While the claims that Phospho-Energon made might seem far-fetched and overtly spurious, they resonated deeply with a public grappling with the anxieties and uncertainties of a rapidly changing world, characterised by swift social transformations, technological advancements, and evolving gender roles. In this context, Phospho-Energon offered not just a cure, but a promise of stability, control, and self-improvement, reflecting a significant moment in the history of consumer health products. It is important to recognise that the appeal of such products extended beyond their supposed medicinal benefits; they provided a sense of agency at a time when individuals were increasingly pressured to conform to ideals of productivity, health, and national duty. This rhetoric of self-care as a moral obligation can still be found in modern wellness culture, where individuals are encouraged to take personal responsibility for their health, often through unregulated supplements and alternative remedies. This study of Phospho-Energon, therefore, stands as a reminder of the enduring allure of quick fixes and the powerful influence of marketing in shaping public perceptions of health. The findings also invite us to question how contemporary health practices may be shaped by similar forces and challenges us to critically evaluate the claims made by modern-day equivalents of patent medicines.

## Footnotes

1 Petteri Pietikainen, *Neurosis and Modernity: The Age of Nervousness in Sweden* (Leiden: Brill, 2007), 1.

2 Cited in Marijke Gikswijt-Hofstra, “Introduction: Cultures of Neurasthenia: From Beard to the First World War,” in *Cultures of Neurasthenia: From Beard to the First World War*, ed. Marijke Gikswijt-Hofstra and Roy Porter (Leiden: Brill, 2005), 79; Karin Johannisson, “Bot för en nervtrött generation: ett läkemedel i idéhistorisk belysning,” in *Vetenskap och läkemedel: ett historiskt perspektiv* (Umeå: Lärdomshistoriska samfundet, 1987), 77-112.

3 Jeremy Agnew, *Healing Waters: A History of Victorian Spas* (Jefferson: McFarland, 2019), 93.

4 Lauren Alex O’Hagan, “Packaging Inner Peace: A Sociohistorical Exploration of Nerve Food in Great Britain,” *Food and History* 17 (2019): 183-222.

5 Phospho-Energon Advertisement, *Svenska Dagbladet*, 8 February 1930, 8; “Aeskulapii näring i fabriksform,” *Svenska Dagbladet*, 27 December 1925, 25.

6 Per Ledin and David Machin, *Doing Visual Analysis* (London: SAGE, 2018); Per Ledin and David Machin, *Introduction to Multimodal Analysis* (London: Bloomsbury, 2020).

7 Roy Porter, *A Social History of Madness: The World Through the Eyes of the Insane* (London: Weidenfeld and Nicolson, 1987), 177.

8 For patent medicines, see James Harvey Young, *The Toadstool Millionaires: A Social History of Patent Medicines in America before Federal Regulation* (Princeton: Princeton University Press, 1961); James Harvey Young, *The Medical Messiahs: A Social History of Health Quackery in 20*^*th*^*-Century America* (Princeton: Princeton University Press, 1967); Roy Porter, *Health for Sale: Quackery in England, 1660–1850* (Manchester: Manchester University Press, 1989); M.W. Weatherall, “Making Medicine Scientific: Empiricism, Rationality, and Quackery in Mid-Victorian Britain,” *Social History of Medicine* 9 (1996): 175-194; Roy Porter, *Quacks: Fakers and Charlatans in Medicine* (London: NPI Group, 2000); Michael Brown, “Medicine, Quackery and the Free Market: The ‘War’ against Morison’s Pills and the Construction of the Medical Profession, c. 1830–c. 1850,” in *Medicine and the Market in England and its Colonies, c. 1450–c. 1850*, ed. Mark R.S. Jenner and Patrick Wallis (London: Palgrave Macmillan, 2007), 238-261; Eric W. Boyle, *Quack Medicine: A History of Combating Health Fraud in Twentieth-Century America* (New York: Bloomsbury, 2013). For neurasthenia, see Francis George Goslin, *Before Freud: Neurasthenia and the American Medical Community, 1870–1910* (Urbana: University of Illinois Press, 1987); Janet Oppenheim, *Shattered Nerves: Doctors, Patients and Depression in Victorian England* (Oxford: Oxford University Press, 1991); Gikswijt-Hofstra and Porter, *Cultures of Neurasthenia*; Tracey Loughran, “Hysteria and Neurasthenia in Pre-1914 British Medical Discourse and in Histories of Shell-Shock,” *History of Psychiatry* 19 (2008): 25-46; Marc-Antoine Crocq, “A History of Anxiety: From Hippocrates to DSM,” *Dialogues in Clinical Neuroscience* 7 (2015): 319-325; Anna Katharina Schaffner, *Exhaustion: A History* (New York: Columbia University Press, 2017).

9 Ben Shephard, *A War of Nerves: Soldiers and Psychiatrists in the Twentieth Century* (Cambridge, MA: Harvard University Press, 2001); John Crellin and Dennis B. Worthen, *A Social History of Medicines in the Twentieth Century: To Be Taken Three Times a Day* (New York: Pharmaceutical Products Press, 2004); Denise Maines, “Why the Appeal? A Study of Almanacs Advertising Dr. Chase’s Patent Medicines, 1904–1959,” *Canadian Pharmacists Journal* 145 (2012): 180-185; Jill Kirby, *Feeling the Strain: A Cultural History of Stress in Twentieth-Century Britain* (Manchester: Manchester University Press, 2019).

10 For example, Karin Johannisson, “Bot för en nervtrött generation”; Karin Johannisson, *Medicinens öga: Sjukdom, medicin och samhälle - historiska erfarenheter* (Stockholm: Morstedts, 2013).

11 Pietikainen, *Neurosis and Modernity.*

12 O’Hagan, “Packaging Inner Peace.”

13 Lauren Alex O’Hagan, “The Golden Path to Health”: Marketing Postum as a Cure for Coffee in Early Twentieth-Century Sweden,” *Food, Culture and Society* 27 (2023): 866-888.

14 Oppenheim, *Shattered Nerves*.

15 Pietikainen, *Neurosis and Modernity*, 47.

16
*Nervförslappning* can be found in “Orsakerna till nervositet (nevrasteni) hos oss,” *Hälsovännen* 14 (1899): 373-374. For “nerve fatigue,” see Karin Johannisson, *Nostalgia: en känslas historia* (Stockholm: Bonniers, 2001).

17 Articles by other physicians of the time include W–d [signature for physician Wilhelm Wretlind], “Vår skolungdom och nervositeten,” *Hälsovännen* 20 (1905): 173-177; Henrik Berg, *Läkarebok. Fjärde upplagan. Del 1–2* (Göteborg: Nordiska Förlags AB, 1919), 1318-1319. For “shifting malady,” see Edward Shorter, *From Paralysis to Fatigue: A History of Psychosomatic Illness in the Modern Era* (New York: Free Press, 1992). For “transient mental illness,” see Ian Hacking, *Mad Travelers: Reflections on the Reality of Transient Mental Illnesses* (Cambridge, MA: Harvard University Press, 2002). For an overview on neurasthenia, see Matthew Thomson, “Neurasthenia in Britain: An Overview,” in *Cultures of Neurasthenia*.

18 Pietikainen, *Neurosis and Modernity*, 4.

19 O’Hagan, “Packaging Inner Peace,” 187.

20 Richard von Krafft-Ebing, *Om friska och sjuka nerver* (Stockholm: J. Seligmann & K, 1885); Paolo Montagazza, *Vårt nervösa århundrade* (Stockholm: Geber, 1888); Willy Hellpach, *Nervositet och kultur* (Stockholm: Geber, 1904).

21 Pietikainen, *Neurosis and Modernity*, 309.

22 Hjalmar Selldén, ”Om vår tids predikant-sjuka,” *Hälsovännen* 10 (1895): 210.

23 Berg, *Läkarebok*, 1318-1320.

24 On different categories of patent medicines on the Swedish market, see Leif Runefelt, *Den magiska spegeln. Kvinnan och varan i pressens annonser 1870–1914* (Lund: Nordic Academic Press, 2019), 165-174; Roger Qvarsell, ”Den sjukes skärv: Medicinsk reklam på en växande marknd före 1900,” in *Reklam och hälsa: Levnadsideal, skönhet och hälsa i den svenska reklamens historia*, ed. Roger Qvarsell and Ulrika Torell (Stockholm: Carlssons, 2004), 30-56; Göran Eriksson and Lauren Alex O’Hagan, “Selling ‘Healthy’ Radium Products with Science: A Multimodal Analysis of Marketing in Sweden, 1910–1940,” *Science Communication* 43 (2021): 740-767.

25 See Judy Z. Segal, “The Empowered Patient on a Historical-Rhetorical Model: 19th-century Patent-Medicine Ads and the 21st-century Health Subject,” *Health* 24 (2019): 572-588.

26 ”Breflåda,” *Hälsovännen* 10 (1895): 107; *Hälsovännen* 15 (1900): 314; quote from ”Breflåda,” *Hälsovännen* 17 (1902): 384. See also ”Breflåda,” *Hälsovännen* 19 (1904): 48; *Hälsovännen* 27 (1912): 98.

27 ”Nervös,” *Svensk Damtidning* 12 (1901): 21; ”’Den moderna kvinnan är till dels en invalid,’” *Svensk Damtidning*, 287; ”Spridda drag från en stora internationella kvinnokongressen i Berlin. III,” *Svensk Damtidning* 15 (1904): 332; ”Insända böcker,” *Svensk Damtidning* 17 (1906): 276-277.

28 Pietikäinen, *Neurosis and Modernity*, 345.

29 Bror Gadelius, ”Om vår tids sinnessjukvård och den allmänna meningen,” *Förhandlingar*, 26 February 1907, 44.

30 Qvarsell, ”Den sjukes skärv,” 48-50; Johannisson, “Bot för en nervtrött generation”; Bosse Sundin, “Universalsmörjelsen. Kvacksalveri, patentmedicin och drömmen om ett naturligt levnadssätt i slutet av 1800-talet,” *Lychnos* 52 (1988): 96-97.

31 Torsten Thunberg, *Tidningarna och reklammedicinen* (Lund: Håkan Ohlssons boktryckeri, 1919); Sundin, *Universalsmörjelsen*, 91-112; *Betänkande med förslag till lagstiftning om vitaminiserade livsmedel och om kringföringshandel med kött och charkuterivaror* (Stockholm: Socialdepartementet, 1937), 51.

32 Pietikainen, *Neurosis and Modernity*, 75.

33 Prop. 1915:85 (’med förslag till förordning om behörighet att utöva läkarkonsten’), https://lagen.nu/prop/1915:85?attachment=index.pdf&repo=propkb&dir=downloaded; Ulf Bernitz and John Draper, *Consumer Protection in Sweden: Legislation, Institutions and Practice* (Stockholm: Institute for Intellectual Property and Market Law, 1986).

34 Johannisson, “Bot för en nervtrött generation”; Runefelt, *Den magiska spegeln*, 169-176. For Sanatogen on the global market, see Lesley Steinitz, “Transforming Pig’s Wash into Health Food: The Construction of Skimmed Milk Protein Powders,” *Global Food History* 9 (2023): 290-323.

35 Advertisement for Neurasthenin, Albinzons Fabriker, *Dagens Nyheter*, 28 July 1906.

36 Oppenheimer, *Shattered Nerves*, 99.

37 Pietikainen, *Neurosis and Modernity*, 187.

38 Johannisson, “Bot för en nervtrött generation.”

39 Lauren Alex O’Hagan, “From Fatigue Fighter to Heartburn Healer: The Evolving Marketing of a Functional Beverage in Sweden,” *Journal of Food Products Marketing* 29 (2023): 19-40.

40 O’Hagan, “Packaging Inner Peace.”

41 Advertisement for Energon, Pharmacia, *Dagens Nyheter*, 28 August 1912.

42 A.K., ”Orsaker till nervositet,” *Hälsovännen* 31 (1916): 12-13.

43 ”Breflåda,” *Hälsovännen* 32 (1917): 304.

44 Advertisment for Energon-produkter, *Dagens Nyheter*, 16 November 1913.

45 Advertisement for phosphate-powder, Tekniska Fabriken Röda Korset, *Hälsovännen*, 1 January 1893; Advertisement for phosphate-powder for children, Caroline af Ekenstam, *Hemmets vän*, 30 December 1903;”Fosfatpulver för barn,” *Hemmets vän*, 18 February 1903.

46 Steinitz, “Transforming Pig’s Wash,” 292. See, for instance, advertisement for Sanatogen, *Hälsovännen*, 16 March 1906.

47 Johannisson, “Bot för en nervtrött generation,” 79-80. The method is explained in a patent for the Swedish market, granted to de Kunwald by the Swedish Patent Office on 15 May 1910, patent nr. SE30532, https://tc.prv.se/spd/patent?p2=2cVmXZSEHOE&content=SE30532&lang=sv&tab=1&hits=true&hitsstart=0&start=0.

48 See for instance, “Breflåda,” *Hälsovännen*, 1 December 1906.

49 Johannisson, “Bot för en nervtrött generation,” 78-79.

50 For similar findings with Postum, see Lauren Alex O’Hagan, “Welcome to Pure Food City”: Tracing Discourses of Health in the Promotional Publications of the Postum Cereal Company, 1920–1925,” *Journal of Historical Research in Marketing* 15 (2023): 171-200.

51 Karin Johannisson, *Den mörka kontinenten. Kvinnan, medicinen och fin-de-siècle* (Stockholm: Norstedts, 1994), 37-56; Runefelt, *Den magiska spegeln*, 163-174.

52 Petrén, see above. “Huru blifva vår tids affärsmän nervösa?” *Hälsovännen* 14 (1899): 256-260; “Från läkarens mottagningsrum. Nervositet,” *Hälsovännen* 17 (1902): 110-112.

53 See O’Hagan, “From Fatigue Fighter to Heartburn Healer” for similar findings with Samarin.

54 Ledin and Machin, *Doing Visual Analysis*, 99.

55 See Lauren Alex O’Hagan, “Blinded by Science? Constructing Truth and Authority in Early Twentieth-Century Virol Advertisements,” *History of Retailing and Consumption* 7 (2021): 162-192 for similar findings with Virol.

56
*Svenska Dagbladet,* 11 February 1935.

57
*Svenska Dagbladet,* 27 September 1938.

58
*Svenska Dagbladet,* 11 October 1938; *Svenska Dagbladet,* 19 September 1939; *Svenska Dagbladet,* 26 September 1939; *Svenska Dagbladet,* 8 November 1933, respectively.

59
*Svenska Dagbladet,* 12 February 1930; *Svenska Dagbladet,* 3 October 1936; *Svenska Dagbladet,* 11 February 1935, respectively.

60
*Svenska Dagbladet,* 10 November 1933; *Svenska Dagbladet,* 7 February 1935, respectively.

61
*Svenska Dagbladet,* 8 March 1933.

62 Tristan Jacques, “On the Road to the Automobile Age Sweden 1923–1973” (MA thesis, Lund University, 2011).

63
*Svenska Dagbladet,* 8 March 1933.

64 Bob Horn, *Visual Language* (Washington DC: Macro VU, 1998).

65 Ledin and Machin, *Doing Visual Analysis*, 134.

66 Ledin and Machin, “Strategic Diagrams and the Technologization of Culture,” *Journal of Language and Politics* 14 (2016): 322-336.

67 Ibid.

68 O’Hagan, “Packaging Inner Peace.”

69
*Svenska Dagbladet*, 31 January 1935; *Svenska Dagbladet,* 5 March 1933, respectively.

70
*Svenska Dagbladet*, 13 November 1933; *Svenska Dagbladet*, 26 February 1935.

71
*Svenska Dagbladet*, 26 February 1935.

72 Sara Thornton, *Advertising, Subjectivity and the Nineteenth-Century Novel* (London: Palgrave Macmillan, 2009), 65.

73
*Svenska Dagbladet*, 19 February 1935; *Svenska Dagbladet,* 6 November 1933, respectively.

74 For similar findings with radium-based products and cod liver oil, see Eriksson and O’Hagan, “Selling ‘Healthy’ Radium Products with Science”; Lauren Alex O’Hagan and Göran Eriksson, “Modern Science, Moral Mothers, and Mythical Nature: A Multimodal Analysis of Cod Liver Oil Marketing in Sweden, 1920–1930,” *Food and Foodways* 30 (2022): 231-260.

75
*Svenska Dagbladet*, 8 February 1930; *Svenska Dagbladet*, 15 February 1930, respectively.

76 Oscar von Friedrichs, “Thor Emanuel Ekecrantz,” *Svenskt biografiskt lexikon*, https://sok.riksarkivet.se/sbl/Presentation.aspx?id=16810.

77 Theo van Leeuwen, “Legitimation in Discourse and Communication,” *Discourse & Communication* 1 (2007): 94.

78
*Svenska Dagbladet*, 8 February 1930.

79 Claire Louise Jones, “(Re-)Reading Medical Trade Catalogs: The Uses of Professional Advertising in British Medical Practice, 1870–1914,” *Bulletin of the History of Medicine* 86 (2012): 391.

80
*Svenska Dagbladet*, 22 October 1935.

81 For more on role model authority, see van Leeuwen, “Legitimation in Discourse and Communication,” 94. Also see Hannah Barker, “Medical Advertising and Trust in Late Georgian England,” *Urban History* 36 (2009): 396.

82
*Svenska Dagbladet*, 5 March 1933.

83 Nick Cullather, “The Foreign Policy of the Calorie,” *American Historical Review* 112 (2007): 337-364.

84
*Svenska Dagbladet*, 17 February 1936 (traffic police); 24 February 1936 (bus driver).

85
*Svenska Dagbladet*, 24 February 1936.

86 Lauren Alex O’Hagan, “Selling Swedish Summer: The Marketing of Pommac, 1920–1960,” *History of Retailing and Consumption* 8 (2022): 171-199.

87
*Svenska Dagbladet*, 19 February 1934.

88 Lovisa Broström, “The Neglected Female Unemployment in Sweden during the Great Depression,” *Labor History* 60 (2019): 704-715; Rebecca Adami, “The Swedish Middle Way and UN Experiences in Domestic Politics: Exploring International Welfare Feminism during Early Cold War Years,” *NORA* 30 (2022): 20-34.

89
*Svenska Dagbladet*, 2 February 1939 (a housewife); *Svenska Dagbladet,* 18 October 1938 (a mother); *Svenska Dagbladet,* 16 November 1937 (a typist); *Svenska Dagbladet,* 17 October 1939 (a maid); *Svenska Dagbladet,* 9 November 1937 (a photograph of a woman; the text states that she works in a store).

90 “Kroppsarbete såsom kur mot nervositet och blodbrist hos kvinnor,” *Hälsovännen* 14 (1999): 191-196; Henrik Berg, *Läkarebok*, 1320-1321; Selldén, ”Om vår tids predikant-sjuka,” *Hälsovännen* 10 (1895): 211-212.

91 Pietikainen, *Neurosis and Modernity*, 257.

92 Victor Wigert, ”Den psykiska hälsan måste värdas!” *Social-Medicinsk Tidskrift* 9 (1932).

93 Gikswijt-Hofstra, “Introduction.”

94 Gadelius, ”Om vår tids.”

95
*Svenska Dagbladet*, 24 February 1936; *Svenska Dagbladet*, 25 October 1938; *Svenska Dagbladet*, 17 October 1939.

96 See Eriksson and O’Hagan, “Selling ‘Healthy’ Radium Products with Science” for similar findings with radium-based products.

97
*Svenska Dagbladet*, 5 March 1936.

98 Shiwaji Pawar and Ashir Kumar, “Issues in the Formulation of Drugs for Oral Use in Children: Role of Excipients,” *Pediatric Drugs* 4 (2012): 371-379.

99
*Svenska Dagbladet*, 9 November 1937.

100
*Svenska Dagbladet*, 16 November 1937.

101
*Svenska Dagbladet*, 6 November 1933.

102
*Svenska Dagbladet*, 10 November 1933.

103 Ibid.

104 Ina Zweiniger-Bargielowska, “Building a British Superman: Physical Culture in Interwar Britain,” *Journal of Contemporary History* 41 (2006): 596.

105 Lesley Steinitz, “The Language of Advertising: Fashioning Health Food Consumers at the Fin De Siècle,” in *Food, Drink, and the Written Word in Britain, 1820–1945*, ed. M. Addyman, L. Wood, and C. Yiannitsaros (London: Routledge, 2017), 135-163.

106
*Svenska Dagbladet*, 13 November 1937. Italics in the advertisement.

107
*Svenska Dagbladet*, 25 October 1938.

108 “Lag om Semester,” *Svensk författningssamling* 1938, 287.

109 O’Hagan, “From Fatigue Fighter to Heartburn Healer.”

110
*Svenska Dagbladet*, 27 February 1939.

111
*Svenska Dagbladet*, 20 March 1940.

112 One of the earliest mentions is in a book review, “Våren – just nu,” *Dagens Nyheter*, 15 November 1898.

113 Linda Wasmer Andrews. “When Spring Brings You Down,” *Psychology Today*, 28 March 2012, https://www.psychologytoday.com/intl/blog/minding-the-body/201203/when-spring-brings-you-down.

114 M.A. Rishi, O. Ahmed, J.H. Barrantes Perez, J. Berneking, E.E. Flynn-Evans, and I. Gurubhagavatula, “Daylight Saving Time: An American Academy of Sleep Medicine Position Statement,” *Journal of Clinical Sleep Medicine* 16 (2020): 1781-1784.

115 ”Hvarpå beror tröttheten om våren?” *Hälsovännen* 14 (1899): 156.

116 A case in point is an article in *Svenska Dagbladet* on 11 March 1906, which reports that a group of judges is unable to work properly because they are “extremely burdened” by springtime lethargy.

117
*Svenska Dagbladet*, 25 April 1926.

118 Kress and van Leeuwen, *Reading Images*, 154.

119 For more on inconguence, see T. Swenberg and P.E. and Eriksson. “Effects of Continuity or Discontinuity in Actual Film Editing,” *Empirical Studies of the Arts* 36 (2018): 222-246.

120 Mark W. Weatherall, “Making Medicine Scientific: Empiricism, Rationality, and Quackery in mid-Victorian Britain,” *Social History of Medicine* 9 (1996): 175-194.

121 For more on this in the context of the cough drop Läkerol, see Lauren Alex O’Hagan and Göran Eriksson, “Blurring the Boundaries Between Medicine and Food: The Canny Marketing of Läkerol in Early Twentieth-Century Sweden,” *Social History of Medicine* (2024), https://doi.org/10.1093/shm/hkae038.

122 Kress and van Leeuwen, *Reading Images*, 65.

123 Ledin and Machin, *Introduction to Multimodal Analysis*, 182.

124 Ledin and Machin, *Doing Visual Analysis*, 134.

125 Ledin and Machin, *Introduction to Multimodal Analysis*, 182.

126 Roland Barthes, *Image-Music-Text* (London: Fontana, 1977), 38.

127 David Machin and Andrea Mayr, *How to Do Critical Discourse Analysis: A Multimodal Introduction* (London: SAGE, 2012).

128
*Svenska Dagbladet*, 30 March 1940.

129 O’Hagan, “From Fatigue Fighter to Heartburn Healer.”

130
*Svenska Dagbladet*, 18 April 1940; *Svenska Dagbladet*, 28 March 1940, respectively.

131
*Svenska Dagbladet*, 18 April 1940; *Svenska Dagbladet*, 22 April 1940; *Svenska Dagbladet*, 18 April 1940, respectively.

132 See E. El Refaie, *Visual Metaphor and Embodiment in Graphic Illness Narratives* (Oxford: Oxford University Press, 2019); Lori A. Loeb, *Consuming Angels: Advertising and Victorian Women* (Oxford: Oxford University Press, 1994).

133 Kirby, *Feeling the Strain*, 40.

134 For similar findings with Chymol, see Lauren Alex O’Hagan, “Commercialising Public Health During the 1918–19 Spanish Flu Pandemic in Britain,” *Journal of Historical Research in Marketing* 13 (2021): 161-187.

135
*Svenska Dagbladet*, 30 March 1940.

136 G. Scrinis, *Nutritionism: The Science and Politics of Dietary Advice* (New York: Columbia University Press, 2013), 42.

137 “Lagstiftning om vitamisering av livsmedel,” *Statens offentliga utredningar* 1937, 51.

138 R.H. Anderson, “Breakfast Cereals and Dry Milled Corn Products,” in *Iron Fortifications of Foods*, ed. Fergus M. Clydesdale and Kathryn L. Wiemer (Orlando: Academic Press, 1985), 116.

139
*Svenska Dagbladet*, 26 March 1942.

140 ”Vårluften söker” in Swedish.

141 Gunther Kress and Theo van Leeuwen, “Colour as a Semiotic Mode: Notes for a Grammar of Colour,” *Visual Communication* 1 (2002): 348.

142
*Svenska Dagbladet*, 4 April 1942.

143 Erin Elizabeth Bramwell, ”’She Used to Doctor Us up Herself’: Patent Medicines, Mothers, and Expertise in Early Twentieth-Century Britain,” *Twentieth Century British History* 31 (2020): 555-578.

144 S.H. Zeisel, “A Brief History of Choline,” *Annals of Nutrition & Metabolism* 61 (2012): 254-258.

145
*Svenska Dagbladet*, 22 April 1950.

146 Pietikainen, *Neurosis and Modernity. Folkhemmet* [The People’s Home] is a term used to describe the Swedish welfare state, implemented by the Swedish Social Democratic Party during their long uninterrupted time in power from 1932 to 1976.

